# Editorial Note: A risk-averse sustainable perishable food supply chain considering production and delivery times with real-world application

**DOI:** 10.1371/journal.pone.0354431

**Published:** 2026-07-27

**Authors:** The PLOS One Editors

The *PLOS One* Editors issue this Editorial Note for this article [1] because of concerns related to the peer review process. Readers are encouraged to approach the article [1] with careful consideration. In following up on this matter, PLOS did not evaluate the article’s integrity or scientific validity.

The Editors do not have concerns regarding the authors’ conduct during the peer review process.
